# s_mmpbsa: A Lite and Cross-Platform MM-PBSA Program

**DOI:** 10.3390/molecules31101683

**Published:** 2026-05-15

**Authors:** Jiaxing Zhang, Tao Gu, Chuanxi Li, Wei Qi

**Affiliations:** 1 State Key Laboratory of Chemical Engineering and Low-Carbon Technology, School of Chemical Engineering and Technology, Tianjin University, Tianjin 300072, China; qiwei@tju.edu.cn; 2 School of Engineering, Westlake University, Hangzhou 310014, China; gutao@westlake.edu.cn; 3 Petrochemical Research Institute, PetroChina, Beijing 102206, China; lichuanxi010@petrochina.com.cn; 4 Tianjin Key Laboratory of Membrane Science and Desalination Technology, Tianjin University, Tianjin 300072, China

**Keywords:** molecular interactions, binding energy, molecular dynamics, MM-PBSA, electrostatic screening, interaction entropy

## Abstract

Molecular mechanics/the Poisson–Boltzmann surface area (MM-PBSA) is a popular method for binding energy estimation. Several programs have been developed for performing MM-PBSA calculations in conjunction with Gromacs, the most popular molecular dynamics (MD) software. However, current programs are limited to Linux-based systems and lack cross-platform usability. To address this, we present s_mmpbsa, a lite and cross-platform MM-PBSA program, to support binding energy calculation on native Windows platforms without a subsystem. By incorporating electrostatic screening and interaction entropy, s_mmpbsa achieves improved binding free energy calculation accuracy, validated on a dataset of HIV-1 protease inhibitor complexes. In addition, s_mmpbsa achieves enhanced performance with g_mmpbsa in the same parameters and conditions. Indeed, s_mmpbsa offers an efficient and practical solution for interaction energy calculation from MD simulations in Gromacs, providing valuable protocols for further molecular design applications such as computational enzyme design and molecular screening.

## 1. Introduction

Molecular binding energy provides a direct measure of molecular interactions and plays a key role in studying the properties and functions of molecular systems [1,2]. It has been increasingly applied to large-scale biomolecular systems for purposes including enzyme rational design, structure-based drug design, antibody design, and molecular recognition [3,4,5,6,7,8,9,10,11,12,13]. Thus, accurate interaction energy calculation is a key task in chemistry and life sciences.

**Figure 1:**
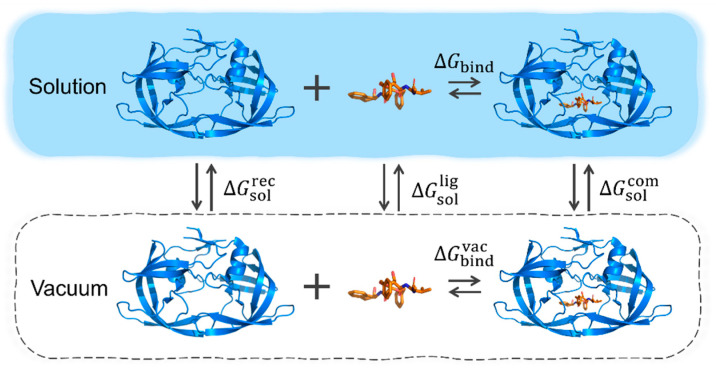
Thermodynamic cycle for MM-PBSA calculation. It is used to approximate the free energy of a solvated system by averaging gas-phase molecular mechanics energies and implicit solvation free energies (calculated via the Poisson–Boltzmann equation and surface area term) over snapshots from an MD simulation, with an optional correction for entropic changes.

**Figure A1:**
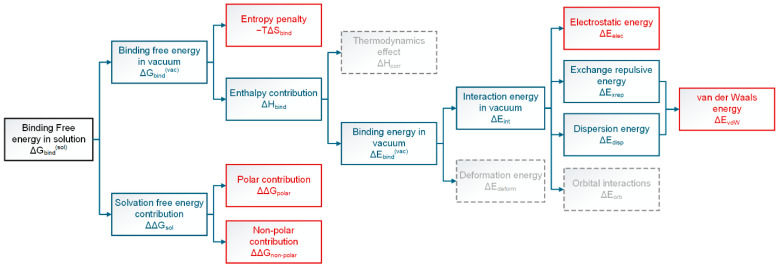
Composition of intermolecular binding free energy. The nodes with gray edge were not considered by s_mmpbsa, and the nodes with red edge were directly given in the final calculation results.

The binding energy within a molecular system in a solution is composed of vacuum binding energy and solvation energy change (Figure 1 and Figure A1). The solvation energy change reflects the influence of solvent effect on intermolecular binding and can be divided into polar and non-polar parts. Vacuum binding energy can be decomposed as electrostatic energy, exchange repulsive energy, and dispersion energy for convenience. Van der Waals energy is the summation of dispersion energy and exchange-repulsion energy, and the binding energy in a solution can be obtained by the summation of the following items: electrostatic energy (ΔE_elec_), van der Waals energy (ΔE_vdW_), polar solvation energy (ΔΔG_polar_), and non-polar solvation energy (ΔΔG_non-polar_). Meanwhile, the interaction entropy method [14] enables us to obtain the entropy penalty term (−TΔS_bind_), which reflects a substantial entropy reduction upon intermolecular binding. With this term determined, the binding free energy can be calculated accordingly. The theoretical background and computational details are provided in Appendix A, Section 3, and the Supplementary Materials.

To obtain binding energy, a variety of computational methods have been developed, including path-dependent methods like Free Energy Perturbation (FEP) [15], Thermodynamic Integration (TI) [16], and path-independent methods like Linear Interaction Energy (LIE) [17] and Molecular Mechanics Poisson–Boltzmann (or Generalized Born) Surface Area (MM-PB(GB)SA) [18]. Among the various approaches, the molecular mechanics/Poisson–Boltzmann surface area (MM-PBSA) method has become popular for estimating binding energies in biological systems [19]. MM-PBSA is a widely used end-state method to calculate binding energy with a balance between computational efficiency and accuracy. In addition, it can be accelerated by the machine learning method [20]. Thus, MM-PBSA is suitable for handling high-throughput and large-scale biological systems such as protein/ligand, protein/protein and nucleic acids.

Typically, there are two methods for MM-PBSA calculations: single-trajectory protocol (STP) and multi-trajectory protocol (MTP) [19]. In STP, the trajectories of both the receptor and the ligand are extracted from the trajectory of the complex. Since only the complex needs to be simulated, its computational cost is lower than that of the MTP method. Meanwhile, the internal energy terms (such as bonds, angles, and dihedrals) cancel out completely because these terms are identical in both the bound and unbound states. On the other hand, MTP is a more realistic approach because it considers the trajectories of the complex, receptor, and ligand separately. However, significant conformational changes may lead to substantial errors.

Molecular interactions are usually studied through molecular dynamics (MD) simulations. Popular MD software includes conventional force-field-based Gromacs [21], Amber [22], NAMD [23], CHARMM [24], LAMMPS [25], OpenMM [26], and Sponge [27], and novel artificial intelligence-based torch-MD [28], JAX MD [29], GPUMD [30], AI^2^BMD [31], and MindSPONGE [32]. Among these, GROMACS remains by far the most widely used program as of 2025 [33]. In recent years, several MM-PBSA programs for GROMACS-based MD simulations have seen considerable advances [34,35,36], including GMXPBSA [34], g_mmpbsa [35], and gmx_MMPBSA [36]. However, most of these tools are designed for Linux-based platforms and lack support for native Windows environments.

Here we present s_mmpbsa to improve the convenience of binding energy calculation on both Windows-based and Linux-based operating systems. Drawing upon the construction of g_mmpbsa v3.0.13 [35], we developed s_mmpbsa in Rust language, to natively perform MM-PBSA calculations following STP on both Windows and Linux platforms. Based on MD simulations of 37 HIV-1 protease inhibitor complexes, we demonstrate the quantitative comparable reliability of s_mmpbsa with g_mmpbsa. We then showcase the availability of s_mmpbsa in biochemistry and biotechnology tasks with several cases, including enzyme computational design, and peptide-based lead drug screening. Indeed, s_mmpbsa exhibits potential availability in multi-scenario binding energy-related biochemistry tasks. The program is available at https://github.com/supernova4869/s_mmpbsa.

## 2. Results and Discussion

### 2.1. Development of s_mmpbsa

**Figure 2:**
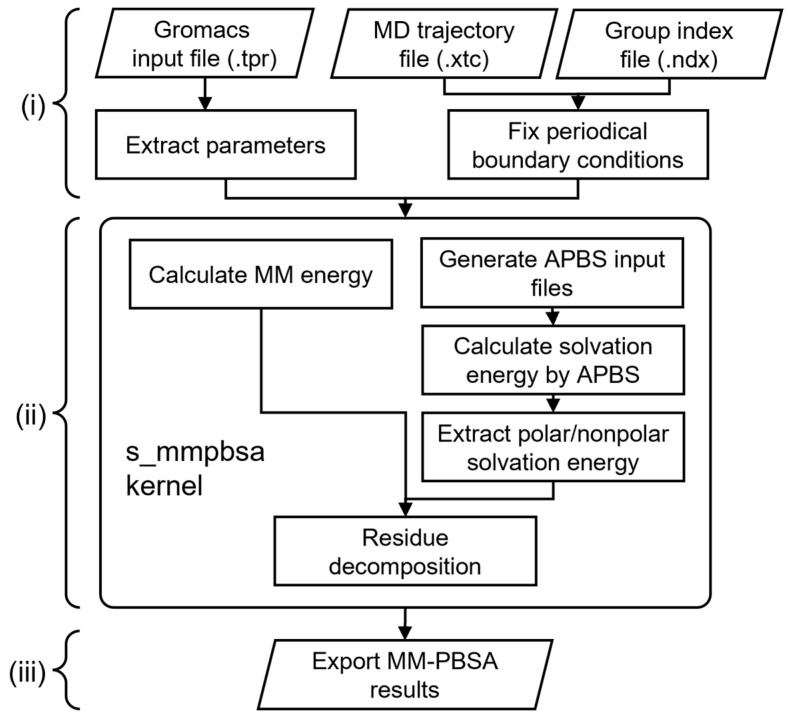
The general workflow of s_mmpbsa: (i) Fix the input trajectory from MD simulation and fix potential periodic conditions; (ii) Calculate vacuum binding energy and solvation energy and decompose to residues; (iii) Analyze and output the calculation results.

The s_mmpbsa program was developed to perform MM-PBSA calculation on the MD trajectory from the Gromacs 2026 simulation package (the version of built-in software will be updated regularly). The implementation scheme of s_mmpbsa for calculating the energy terms is shown in Figure 2, dividing the calculation process into three main steps: (i) preparation, (ii) energy calculation, and (iii) results analysis. The s_mmpbsa program supports cross-platform usage (Linux and Windows), with high accuracy and efficiency.

During program running, the required coordinates and key parameters of the MD simulation such as Lennard–Jones parameters, atomic type, atom charge, system composition, and temperature are obtained from a .tpr file by invoking “gmx dump” utility. The index file will be generated from the .tpr file if the .ndx file is not provided. The periodic boundary condition of the trajectory is fixed by “gmx trjconv” utility. Finally, s_mmpbsa calculates Δ*E*_MM_ based on the above parameters and invokes the built-in APBS 1.5 program [1] to calculate the solvation energy, whose key parameters are set through the input config.yaml file (default template could be generated with “-c” startup option), with the miscellaneous parameters temporally editable by an external .yaml file. The entropy penalty was calculated in parallel.

The analysis results given by the program include the following points:

- Summary of MM-PBSA-related energy terms: ΔH, ΔE_MM_, ΔG_polar_, ΔG_non-polar_, ΔE_elec_, ΔE_vdW_, −TΔS, and ΔG (ΔH is the summation of ΔE_MM_, ΔG_polar_, and ΔG_non-polar_, according to Section 3.1). Standard deviations are provided for all terms except −TΔS and ΔG.
- Changes in the above energy terms (except −TΔS and ΔG) along the trajectory.
- Average of the above energy terms (except −TΔS and ΔG) decomposed to each residue along the trajectory.
- Binding energy decomposed to each atom and shown as B-factor putty mode with user’s PyMOL (optional).

Detailed information of the program can be found in Appendix B.

### 2.2. User Interface

The s_mmpbsa program supports two interface modes: command-line interface (CLI) mode and configuration-driven interface (CDI) mode. In CLI mode, users specify all parameters directly in the program input and output (IO) environment, making it ideal for “ready-to-serve” tests and interactive operations like Multiwfn [37], a wave function analysis program. In CDI mode, users prepare a configuration file with a .yaml format with all parameters and launch the program with a single command, which is particularly suitable for batch processing.

The available input includes the trajectory file (.xtc), the topology parameter file (.tpr), and the optional index file (.ndx). Structural files (.pdb and .gro) can also act as trajectory input. Meanwhile, the CDI mode can be activated by providing the .yaml file with a “-c” option. The program enters analysis mode with an “-a” option. All the available input options are listed in Table 1.

### 2.3. Accuracy and Performance Test

To test the accuracy of s_mmpbsa, we selected a dataset of 37 HIV-1 protease inhibitor complexes previously used with g_mmpbsa [35]. Using both s_mmpbsa and g_mmpbsa, we calculated the binding energies from MD simulations and compared them against the experimental ΔG values derived from inhibition constants (Table S2). To ensure a fair comparison, all calculations were performed under identical simulation conditions (Tables S4 and S5).

**Figure 3:**
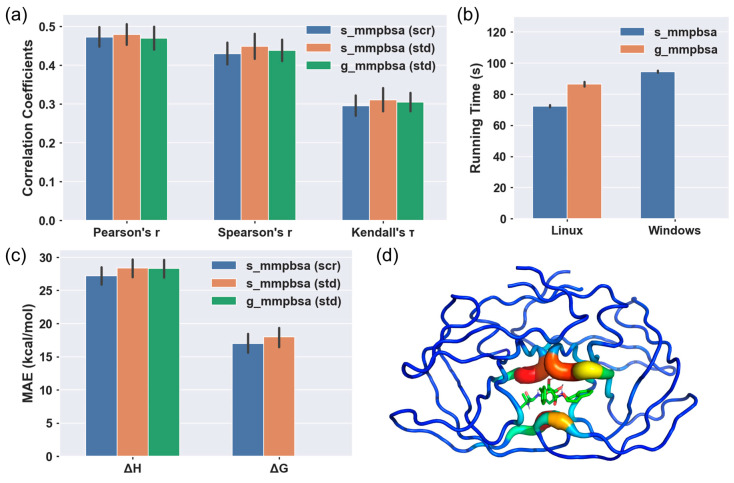
Comparison of s_mmpbsa and g_mmpbsa with ΔG_expr_ under electric screening (scr) and standard calculation (std). (**a**) Pearson correlation coefficient, Spearman correlation coefficient, and Kendall’s *τ* correlation coefficient of the two programs with ΔG_expr_, with electric screening (scr) and standard calculation (std). (**b**) Average calculation time of the two programs on the dataset. (**c**) Mean absolute error (MAE) of ΔH and ΔG calculation of the two programs with ΔG_expr_, with electric screening (scr) and standard calculation (std). (**d**) Binding energy decomposition onto protein residues (1EBZ). The red and thick residues correspond to stronger binding energy contribution, and the blue and thin ones the weaker.

We first compared ΔH from both programs with ΔG_expr_ (Figure 3). We found that the Pearson, Spearman and Kendall’s *τ* correlation coefficients between the ΔH predicted by s_mmpbsa (with and without electric screening) and g_mmpbsa and ΔG_expr_ were calculated to be numerically equal, indicating a strong positive correlation and demonstrating that s_mmpbsa can effectively rank the relative binding affinities of the studied complexes as g_mmpbsa (Figure 3a and Figure S1). Meanwhile, the mean absolute errors (MAEs) between ΔH predicted by s_mmpbsa (with and without electric screening) and g_mmpbsa and ΔG_expr_ are 27.18 (28.37) and 28.30 kcal/mol, respectively, while that between the ΔG predicted by s_mmpbsa (with and without electric screening) and Δ*G*_expr_ is reduced to 16.99 (18.00) kcal/mol (Figure 3c). This shows that s_mmpbsa exhibits considerable reliability and smaller errors with the experimental values. Detailed energy values are listed in Table S3. Indeed, compared with g_mmpbsa, the similar correlation coefficients and reduced MAE illustrate the feasibility of s_mmpbsa to reflect receptor-ligand interaction strengths.

Regarding residue decomposition, s_mmpbsa supports decomposing binding energy to each residue. By filling residue binding energy to a B-factor column, we illustrate the binding energy leveraging the “B-factor putty” mode in PyMOL. The thickness of the cartoon representation is scaled proportionally to the magnitude of the residue’s binding contribution (with thicker tubes indicating stronger energetic contributions), and the color gradient (typically ranging from red to white to blue) reflects the absolute value of the binding energy, where warmer colors (e.g., red) denote residues with highly favorable binding energies, while cooler colors represent neutral or unfavorable contributions (Figure 3d). Key interacting residues, such as those forming hydrogen bonds or hydrophobic contacts at the binding interface, are prominently highlighted as thick, warm-colored tubes. This intuitively visualizes energy data, thereby enabling the rapid identification of key binding residues and facilitating the rational design targeting these hotspot residues.

Finally, we compared the calculation performance of s_mmpbsa and g_mmpbsa (Figure 3b and Figure S2). To exclude the influence of different hardware performances, we performed the comparison on the same Intel i7-12700KF machine, equipped with dual operating systems: Windows 10 and Rocky Linux 10. Based on 8-kernel parallel computations on the Rocky 10 system, s_mmpbsa showed a higher performance than g_mmpbsa on the Linux system, with time costs being 72.19 s and 84.36 s. On the Windows 10 system, s_mmpbsa also exhibits comparable performance with the 8-kernel parallel, with 94.35 s of time cost. Thus, s_mmpbsa showed high performance on binding energy calculations and its usability on large-scale biosystem applications.

### 2.4. Binding Energy Calculation: Case Studies

The s_mmpbsa program has been applied in numerous biochemical studies (Appendix C). Here we give two tasks for case studies: enzyme computational design and amyloid aggregation inhibitor design.

#### 2.4.1. Case 1: Enzyme Computational Design

Enzyme engineering aims to enhance catalytic efficiency. Conventional wet-lab-based enzyme engineering methods are often inefficient and costly. Computational enzyme design offers an effective alternative [38]. However, the success rate of computational design remains limited. To address this, we incorporated s_mmpbsa to calculate the binding energy between each residue of enzyme and its substrate. This energy calculation serves as an in silico filter, allowing us to predict potential promising mutable residues before experimental validation. Consequently, our approach provides a quantitative basis for prioritizing enzyme variants, thereby guiding downstream enzyme engineering efforts toward higher catalytic activity.

**Figure 4:**
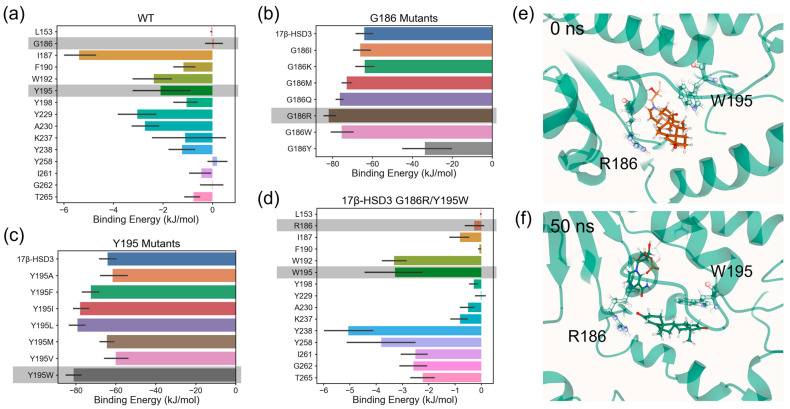
Case study of using s_mmpbsa to enhance 17β-HSD3 enzyme engineering. (**a**) Contribution of each residue in 17β-HSD3’s active pocket to the enzyme–substrate binding energy. Shadowed residues are two identified key residues: G186 and Y195. (**b**,**c**) Change in enzyme–substrate binding energy of each mutant compared to wild-type with substrate. Mutants were obtained by semi-saturation mutagenesis on (**b**) G186 and (**c**) Y195. Shadowed mutations are two identified potential positive mutations: G186R and Y195W. (**d**) Contribution of the same residues as (**a**) in 17β-HSD3’s double mutant (G186R/Y195W) to the enzyme–substrate binding energy. Shadowed mutated residues R186 and W195 showed enhanced contribution to the enzyme–substrate binding energy. (**e**,**f**) The conformations of the double mutant in 50 ns MD simulations at (**e**) 0 ns and (**f**) 50 ns, which showed the mechanism of the double mutant with enhanced activity. R186 and W195 stabilized the substrate by forming new electrostatic and hydrophobic stacking interactions, thereby increasing the enzyme–substrate affinity.

The 17β-hydroxysteroid dehydrogenase 3 enzyme (17β-HSD3) is highly expressed in the male reproductive system. It catalyzes the reduction of substrate 4-AD to testosterone, which is a key male hormone and drug molecule. In previous work [3], we studied the 17β-HSD3 mutants and found that the G186R/Y195W double mutant showed the best catalytic performance through two rounds of wet lab screening. Here we reproduce the computational design protocol by s_mmpbsa (Figure 4). The 17β-HSD3/4-AD complex was established and then subjected to a 40 ns MD simulation. We used s_mmpbsa to analyze the binding energy and decomposed the energy contributions to residues. The two key residues G186 and Y195 were first identified, and then semi-saturated mutations were designed for G186 and Y195, respectively, and the complex structure of each mutant with 4-AD was established, and finally the G186R/Y195W mutant was screened out through MD and MM-PBSA analysis of each mutant.

The results are consistent with the previous experiments that show that G186R/Y195W remains the highest testosterone producer [3]. This work supports s_mmpbsa’s potential to provide ideas for the design of active mutants that enhance enzyme activity.

#### 2.4.2. Case 2: Anti-Aggregation Inhibitor Analysis

Intermolecular binding energy plays a key role in molecular screening, thereby accelerating the development of more efficient bioactive molecules. Computational binding energy prediction offers a more efficient alternative to conventional resource-intensive high-throughput screening methods for obtaining binding affinity. Based on this, we incorporated s_mmpbsa to compute the binding energy between small-molecule ligands and the receptor-binding pocket, thereby facilitating molecular screening.

**Figure 5:**
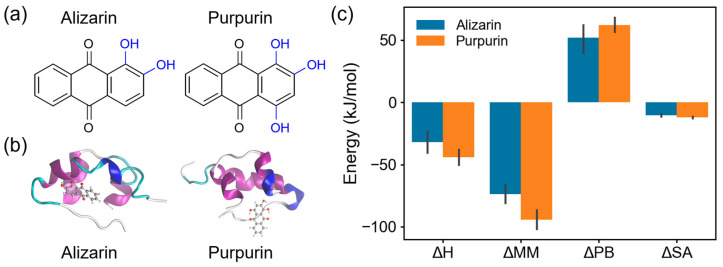
Analysis of the inhibitory effects of alizarin and purpurin molecules on insulin aggregation using s_mmpbsa. (**a**) Molecular structures of alizarin and purpurin. (**b**) Complex structures of both molecules with insulin (PDB ID: 1ZNI). (**c**) Binding energy terms of both molecules with insulin.

In our previous work [39], we selected insulin as a model amyloid protein and compared the inhibition performance of two anthraquinone molecules, alizarin and purpurin, in inhibiting amyloid aggregation (Figure 5). The experimental results showed that purpurin showed a stronger inhibitory effect on insulin aggregation. To explain this phenomenon, we rebuild the structure of the complex of insulin/alizarin and insulin/purpurin and performed a 40 ns MD simulation on each system. We then calculated the binding energy of alizarin/purpurin and insulin by s_mmpbsa and found that the binding energy of purpurin with insulin is stronger than with alizarin. This is because the purpurin molecule has more hydroxyl groups and can form a stronger interaction with insulin and thus has a stronger affinity (lower ΔE_MM_). Therefore, purpurin is a potential amyloid aggregation inhibitor molecule with an efficient inhibitory effect (consistent with our recent study [40]).

## 3. Materials and Methods

### 3.1. Binding Energy Calculation

In general, binding free energy ΔG_bind_ in a solution can be expressed as:

(1) $$\Delta G_{bind}=\Delta H-T\Delta S=\Delta E_{MM}+\Delta G_{sol}-T\Delta S$$

where ΔH is the average energy change in a solution, ΔE_MM_ is the average molecular mechanical potential energy change in vacuum, TΔS is the entropy penalty, and ΔG_sol_ is the average solvation free energy.

#### 3.1.1. Molecular Mechanics Potential Energy

The vacuum molecular mechanics term ΔE_MM_ includes the energy of bonded and non-bonded interactions and is calculated based on the molecular mechanics (MM) force field parameters derived from MD simulations.

(2) $$\Delta E_{MM}=\Delta E_{internal}+\Delta E_{vdW}+\Delta E_{elec}$$

where ΔE_internal_ represents the system internal energy consisting of bonding energy, including bond length, angle, torsion, and improper dihedral terms. Non-bonded interactions include electrostatic interactions (ΔE_elec_) and van der Waals interactions (ΔE_vdW_). Since s_mmpbsa adopts STP, the ΔE_internal_ term between the complex and receptor ligand is canceled out and exactly zero.

#### 3.1.2. Solvation Energy

Solvation energy is the energy required to transfer a solute from a vacuum into a solvent. The implicit solvent model is used for MM-PBSA calculations. Solvation energy is expressed as

(3) $${\Delta G}_{sol}={\Delta G}_{polar}+{\Delta G}_{non-polar}$$

where ΔG_polar_ and ΔG_non-polar_ are the polar and non-polar contributions to solvation energy, respectively. ΔG_polar_ is calculated by solving the Poisson–Boltzmann Equation (PBE):

(4) $$-\nabla \cdot \left[\varepsilon \left(r\right)\nabla \varphi \left(r\right)\right]+\varepsilon \left(r\right){\bar{\kappa }}^{2}\left(r\right)\sinh\left(\varphi \left(r\right)\right)=\frac{4\pi \rho_{f}\left(r\right)}{k_{B}T}$$

where *φ* is the target electrostatic potential function, *ε* is the dielectric constant of the solvent, ${\bar{\kappa }}^{2}$ describes the accessibility of ions to the interior of the solute, *ρ*_f_ describes the distribution of partial charges on the fixed solute atoms, *k*_B_ is the Boltzmann constant, *T* is the temperature, and *r* is the spatial coordinate. For calculation efficiency, when the ion concentration is relatively low (e.g., normal saline of 0.15 mol/L), the nonlinear PBE could be approximated as a linear PBE by approximation sinh(*φ*(r)) ≈ *φ*(r).

The non-polar contribution term ΔG_non-polar_ of dissolution free energy includes the mutual repulsion term and the van der Waals term (detailed in Supplementary Materials). These terms can be estimated by the SASA model, which is one of the most common non-polar models, based on the assumption that the ΔG_non-polar_ term is linearly dependent on the solvent accessible surface area (SASA):

(5) $${\Delta G}_{non-polar}=\gamma A+b$$

where *A* is the SASA of the molecule, and *γ* and *b* are surface tension and bias. Other default parameters have been provided in the Supplementary Materials.

#### 3.1.3. Entropy Penalty

The entropy penalty term is calculated by the interaction entropy method, which can calculate the entropy term directly from the MD simulation without apparent computational cost: [14]

(6) $$-T\Delta S=k_{B}T\ln\left[\frac{1}{N}\sum_{i=1}^{N}e^{\frac{\Delta E_{MM}\left(i\right)}{k_{B}T}}\right]$$

where ΔE_MM_(i) = E_MM_(i) − ⟨E_MM_⟩ represents the fluctuation of protein interaction energy around the average energy E_MM_, *k*_B_ is the Boltzmann constant, and *T* is the temperature.

#### 3.1.4. Electric Screening Correction

Δ*E*_elec_ is the electrostatic interaction energy between the receptor and the ligand. In highly charged systems, the significant net charge of receptors or ligands can lead to substantial ion accumulation, resulting in a pronounced shielding effect. Thus, we implemented an additional exponential decay correction according to the Debye-Huckel theory, as described by Ding et al. [41]:

(7) $$∆E_{elec}=\sum_{i}^{Rec}\sum_{j}^{Lig}\frac{q_{i}q_{j}}{4\pi \varepsilon_{0}\varepsilon_{in}r_{ij}}e^{-r_{ij}/\lambda_{D}}$$

where *q*_i_ and *q*_j_ are the charges of atoms *i* and *j* in the receptor and ligand, respectively, *r*_ij_ is the distance between atoms *i* and *j*, *ε*_0_ is the dielectric constant in vacuum, *ε*_in_ is the relative dielectric constant of the solute, and *λ*_D_ is the Debye length, which can be determined by the Debye–Huckel theory [42]:

(8) $$\lambda_{D}=\sqrt{\varepsilon_{0}\varepsilon_{r}k_{B}T/\sum_{i}c_{i}e^{2}z_{i}^{2}}$$

where *ε*_r_ is the relative dielectric constant of the solvent, *e* is the elementary charge, and *c*_i_ and *z*_i_ are the concentration and net charge of ion i, respectively. Note that *λ*_D_ should be calculated by summarizing all mobile ion species. In normal saline (0.15 mol/L) at 298.15 K (the most usual MD condition), *λ*_D_ = 8.0 Å.

#### 3.1.5. Residue Decomposition

For binding energy decomposition onto residues, interaction energy values of each atom are calculated, and then accumulated per residue:

(9) $$\Delta E_{res}^{bind}=\sum_{i=0}^{n}{\Delta E}_{i}^{atom}$$

where res refers to each residue, E*_i_*^atom^ represents binding energy of each atom within the current residue, and *i* iterates over the *n* atoms of the current residue.

### 3.2. Alanine Scanning

The s_mmpbsa program supports optional in silico alanine scanning, which is a useful strategy in enzyme engineering to identify key residues. If enabled, s_mmpbsa will perform alanine mutations on the selected residues of the enzyme on every frame by removing the side chains atoms of the target residues up to the Cβ atom, fixing H atoms, and then recalculating the energy terms of the new complex structure. The mutated results were exported together with the wild-type (WT) enzyme for further comparation.

### 3.3. Molecular Dynamics Simulation

All the selected complexes were subjected to 10 ns MD simulations. For each HIV-1 protein–ligand system, the ligand coordinates were extracted from the PDB structure file of the complex, and hydrogen atoms were added to the ligand to obtain the complete ligand structure. The protonation state of the protein was determined using the PROPKA server [43], and the structure and topology of the protonated protein were generated using the “gmx pdb2gmx” utility and merged with the ligand structure to form the initial structure of the system. The small molecule topology was generated using the acpype script [44] and merged with the protein topology to form the main topology of the system. The MD parameters of the ligand were derived from the GAFF force field [45].

MD simulations were performed using Gromacs 2025.2 [46]. Each complex was placed at the center of a rectangular periodic box with a margin of 1.5 nm, and filled with TIP3P water molecules [47]. Appropriate amounts of Na^+^ and Cl^−^ at 0.15 mol/L were added to balance the system charge. Energy minimization was performed using the steepest descent algorithm with 2000 steps. The temperature was then raised to 298.15 K over a 100 ps heating period. A 10 ns production phase simulation was performed in an isothermal-isobaric ensemble (NPT) at 298.15 K and 1 bar. The temperature and pressure were controlled using velocity rescale [48] and stochastic cell rescale [49], respectively, with coupling time constants of 0.2 ps and 2 ps, respectively. The cutoff distance for van der Waals forces and short-range electrostatic interactions was 1 nm, and the long-range electrostatic interaction was treated using the smooth Particle Mesh Ewald (PME) method [50]. All bonds were constrained using the parallel LINCS method [51].

### 3.4. Accuracy and Performance Comparation

The experimental ΔG values of the HIV-1 complexes were derived from the inhibition constant (K_i_), which is equivalent to the dissociation constant (K_d_):

(10) $$\Delta G_{expr}=-RT\ln\left(\frac{1}{K_{i}}\right)=-RT\ln\left(\frac{1}{K_{d}}\right)$$

where ΔG_expr_ is the experimental ΔG, and *R* and *T* are the ideal gas constant and temperature, respectively. To ensure the consistency, we used the same HIV-1 protease inhibitor data set [35]; details in the Supplementary Materials.

Pearson’s *r* is calculated as:

(11) $$r=\frac{\sum_{i=1}^{n}\left(X_{i}-\bar{X}\right)\left(Y_{i}-\bar{Y}\right)}{\sqrt{\sum_{i=1}^{n}{\left(X_{i}-\bar{X}\right)}^{2}}\sqrt{\sum_{i=1}^{n}{\left(Y_{i}-\bar{Y}\right)}^{2}}}$$

Spearman’s *r* is calculated as:

(12) $$r=1-\frac{6\sum d_{i}^{2}}{n(n^{2}-1)}$$

where *d*_i_ is the difference between the ranks of each pair of observations, and *n* is the sample size.

Kendall’s *τ* is calculated as:

(13) $$\tau =\frac{2\left(n_{c}-n_{d}\right)}{n(n-1)}$$

where *n*_c_ is the number of concordant pairs, *n*_d_ is the number of discordant pairs, and *n* is the sample size.

For the performance test, we read the information of the native time cost output file of s_mmpbsa, and examined the time cost of g_mmpbsa by the “time” command of the Rocky 10 and Windows 10 system.

## 4. Conclusions

In this study, we developed s_mmpbsa, a lite and cross-platform MM-PBSA program for Gromacs, aiming to efficiently and accurately calculate binding energy. The program incorporates modules for calculating intermolecular interaction energies, polar/non-polar solvation energies, and supports electric screening and interaction entropy, with residue-wised energy decomposition capability. With the HIV-1 protease-inhibitor complex benchmark system, s_mmpbsa exhibited a similar Pearson correlation coefficient and MAE with g_mmpbsa, while the binding free energy showed smaller a MAE. Further applications in enzyme engineering and aggregation inhibitor analysis validated the program’s robustness in complex biochemical systems, establishing s_mmpbsa as a useful tool for biomolecular applications such as computational enzyme redesign, lead drug screening and mechanism studies.

## Figures and Tables

**Table 1:** Startup options of s_mmpbsa. Usage: s_mmpbsa [[options] [parameters]].

Startup Options	Input Parameter	Description
-f	md.xtc/pdb/gro	input trajectory file path
-s	md.tpr	input tpr file path
-n	index.ndx	input index file path
-a, --analyze	example.sm	enter analysis mode
-c, --config	[config.yaml]	assign config file path; if not provided, generate config.yaml at current directory
-v, --version	—	show version info
-h, --help	—	print help

## Data Availability

The source code and configuration instructions are available on GitHub at https://github.com/supernova4869/s_mmpbsa under an MIT license. Detailed documentation and step-by-step usage examples can be found in the README.md file within the repository. Users are encouraged to report issues or suggestions through the GitHub Issues page so they can be addressed promptly. The functionality of the program relies on third-party programs (APBS and PyMOL). The data illustrated in this application note are stored at https://doi.org/10.5281/zenodo.19124068.

## Supplementary Materials

The following supporting information can be downloaded at: https://www.mdpi.com/article/10.3390/molecules31101683/s1, Default settings of s_mmpbsa [52,53,54]; Figure S1: Correlation between experimental ΔG and predicted binding energy; Figure S2: Cost of running time comparison; Table S1: Different atomic radii sets of s_mmpbsa used; Table S2: The PDB entries and inhibition constants (*K*_i_) (in nM) of the 37 HIV-1 protease inhibitor complexes; Table S3: Comparison of binding energy for 37 HIV-1 complexes using s_mmpbsa and g_mmpbsa and experimental data; Table S4: The parameters used in Δ*G*_polar_ calculation; Table S5: The parameters used in Δ*G*_non-polar_ calculation.

## Appendix A. Theory of Molecular Interactions and Binding Energy Calculation

Molecular interactions in solutions mainly manifest in two key parts: molecular interactions in vacuum and solvent effect, departing from entropy penalty.

The main essence of molecular interactions in vacuum includes three fundamental types: electrostatic interaction (shows either repel or attract effects according to atom charge), dispersion interaction (attractive, corresponds to the long-range Coulomb correlation of electrons), and exchange-repulsion (extremely repulsive effect at a very close distance). These three fundamental interactions mix with varying proportions to form common types of molecular interactions, e.g., van der Waals interaction, hydrogen bond, and π-π stacking, which have significant effects on the properties and behaviors of the molecular systems. Particularly, the van der Waals interaction is the union of dispersion interaction and exchange-repulsion. Thus, the summation of electrostatic and van der Waals interactions could describe the weak interactions within a molecular system.

The solvent effect in molecular systems can be divided into polar and non-polar components. The polar component reflects the electrostatic interactions between the solute and solvent, including the polarization of the solute’s electronic distribution by the solvent. The non-polar component accounts for various non-electrostatic interactions between the solute and solvent, including the work required to displace solvent molecules and create a solute cavity, the entropy effects of the solute on solvent molecules, and the van der Waals interactions between solute and solvent.

Indeed, the calculation of binding free energy in solutions requires consideration of the above items: vacuum binding free energy, solvation free energy change, and entropy penalty (Figure A1). The entropy penalty is due to a significant decrease in entropy caused by intermolecular binding. The solvation free energy change reflects the influence of the solvent effect on intermolecular binding and can be divided into polar and non-polar parts. The vacuum binding free energy mainly comprises enthalpy contribution. The enthalpy contribution can be decomposed as electrostatic energy, exchange repulsive energy, and dispersion energy for convenience. Van der Waals energy is the summation of dispersion energy and exchange-repulsion energy, and the binding free energy (ΔG_bind_) in a solution can be obtained by the summation of the following items: electrostatic energy (ΔE_elec_), van der Waals energy (ΔE_vdW_), the solvation part (ΔΔG_sol_), including polar solvation energy (ΔΔG_polar_), non-polar solvation energy (ΔΔG_non-polar_), and the entropy penalty (−TΔS_bind_).

## Appendix B. Program Architecture and Installation

s_mmpbsa operates as a desktop program on both Windows and Linux systems. It was developed with the Rust programming language, invoking the pre-compiled GROMACS and APBS utilities for cross-platform compatibility. The latest GROMACS executable file for MD trajectory pre-treating will be kept updated to ensure support of reading a .tpr file from all previous versions of GROMACS. The installation consists only of downloading the latest program from the release page (https://github.com/supernova4869/s_mmpbsa/releases), uncompressing it, and adding the directory to a PATH environment variable. In setting a .ini file, the user can set the basic parameters of s_mmpbsa, including the related programs path and number of parallel kernels to be used. The molecular mechanics part (ΔE_MM_ = ΔE_elec_ + E_vdW_) is calculated by Rust natively, while the solvation part (ΔΔG_sol_ = ΔΔG_polar_ + ΔΔG_non-polar_) is obtained by the built-in parallel APBS program. The entropy penalty was estimated by the interaction entropy (IE) method [14] and the electric screening correction algorithms were implemented to improve the accuracy of interaction energy calculation [41].

## Appendix C. Publications Utilizing s_mmpbsa

Since its first release, s_mmpbsa has been adopted by an increasing number of studies across diverse fields, including protein engineering, inhibitor design, and biomolecular mechanisms analysis. Here, we partially list the publications until 9 May 2026 that have utilized s_mmpbsa in Table A1.

**Table A1:** Some representative publications utilizing s_mmpbsa.

Journal	Year	Volume(Issue)	Pages	JCR
*Nat. Commun.*	2025	*16*(1)	5060	Q1
*Chem. Mater.*	2025	*37*(18)	7326–7336	Q1
*J. Hazard. Mater.*	2025	*490*(15)	137837	Q1
*Colloids. Surf. B*	2025	*250*	114538	Q1
*J. Hazard. Mater.*	2026	*503*	141130	Q1
*Biomacromolecules*	2026	*27*(1)	964–977	Q1
*ACS. Synth. Biol.*	2026	*15*(1)	284–296	Q1
*Molecules*	2026	*31*	1092	Q2
…	…	…	…	
