# Supplementary material for: s_mmpbsa: A Lite and Cross-Platform MM-PBSA Program
Source: Molecules. 2026 May 15;31(10):1683. doi: 10.3390/molecules31101683 (PMC13209212; doi:10.3390/molecules31101683)
Supplement: Supplementary file 1 [file molecules-31-01683-s001.zip › molecules-4274655-supplementary.pdf]

s_mmpbsa: A Lite and Cross-Platform MM-PBSA Program

Jiaxing Zhang [1,*], Tao Gu [2], Chuanxi Li [3] and Wei Qi [1,4]

[1] State Key Laboratory of Chemical Engineering and Low-Carbon Technology, School of Chemical Engineering and Technology, Tianjin University, Tianjin 300072, China
[2] School of Engineering, Westlake University, Hangzhou 310014, China
[3] Petrochemical Research Institute, PetroChina, Beijing 102206, China
[4] Tianjin Key Laboratory of Membrane Science and Desalination Technology, Tianjin University, Tianjin 300072, China
* Correspondence: zhangjiaxing7137@tju.edu.cn

Default settings of s_mmpbsa

ΔE_{MM} calculation. The vacuum interaction energy is calculated with van der Waals and electric methods. The C6 and C12 parameters will be extracted from the force field within the .tpr input file. The optional electric screening method by Ding *et al.* [1] will be used by default.

ΔG_{polar} calculation. The coordinate boundaries of the complex in each dimension will be expanded to 1.5 times (cfac = 1.5) to obtain a coarse grid boundary for APBS; then the fine grid boundary will be obtained by extending 5 Å in each dimension (fadd = 5) from the coordinate boundaries of the molecular complex. An ionic strength of 0.15 M NaCl and radii of 0.95 Å and 1.81 Å for sodium and chloride ions will be used to be consistent with conventional MD simulations settings. The values of vacuum dielectric constant (vdie) and solvent dielectric constant (sdie) will be set as 1 and 78.4, respectively. The solute (pdie) dielectric constant will be set to 2 for biological systems. Then ΔG_{polar} will be obtained by solving the PBE using built-in parallel APBS by default. For ΔE_{MM} calculation, s_mmpbsa provides five available atomic radius types, including the same radius as the MD force field (ff), Amber, Bondi, mBondi, and mBondi2 (values in **Table S1**) [2, 3], consist with g_mmpbsa. All parameters are listed in **Table S4**.

$\Delta G_{non\text{-}polar}$ calculation. $\Delta G_{non\text{-}polar}$ will be calculated using the solvent accessible surface area (SASA) nonpolar model. The atom type is set as mBondi type. The SASA of the molecule will be obtained by APBS by setting solvent detection radius to 1.4 Å and surface tension to 1 (so that the $\Delta G_{non\text{-}polar}$ results given by APBS is numerically equal to SASA). Subsequently, $\Delta G_{non\text{-}polar}$ is calculated with parameters $\gamma = 0.0226778$ kJ/(mol·Å^2), $b = 3.84982$ kJ/mol. All parameters are listed in **Table S5**.

Figures

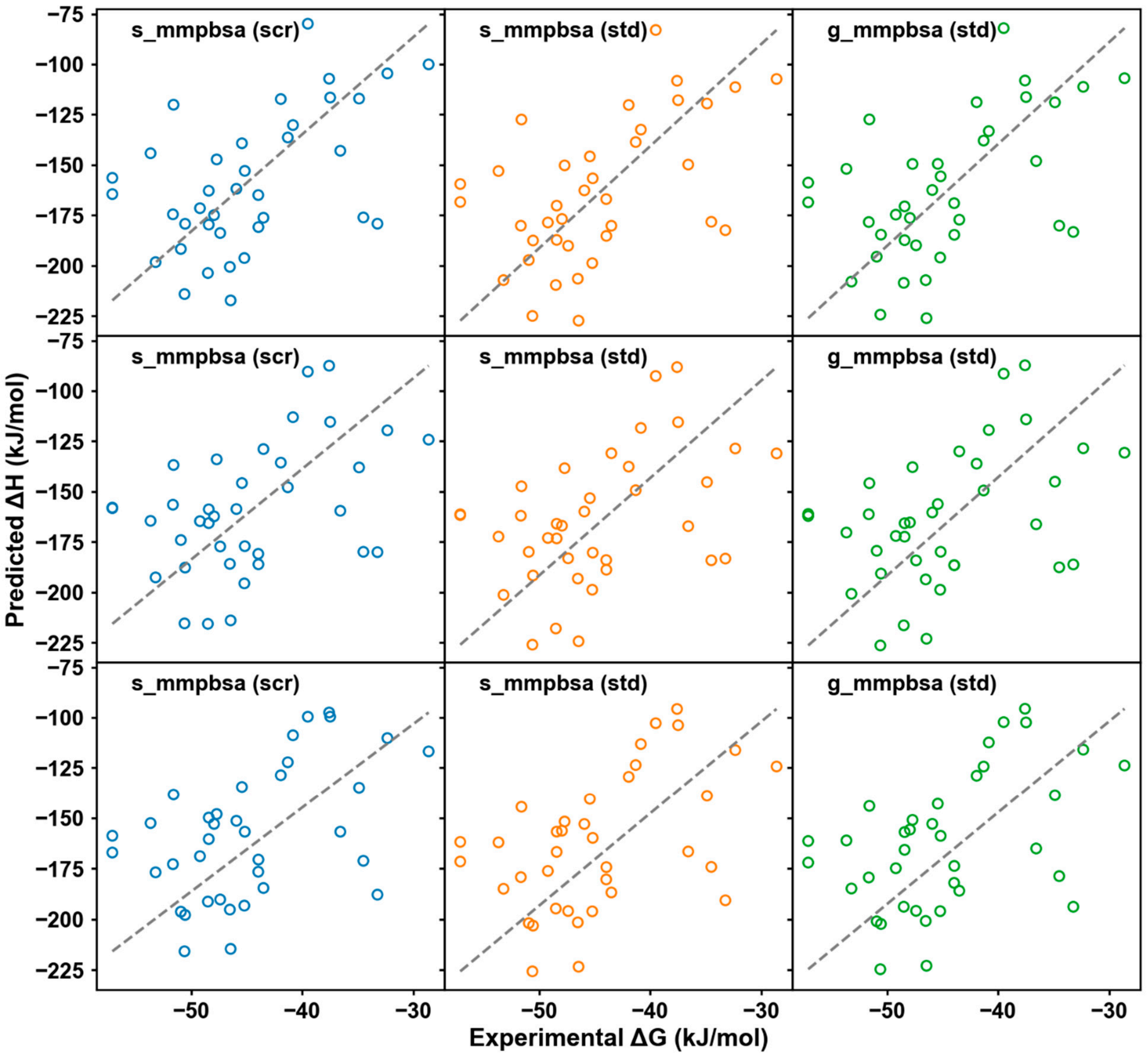

Figure S1 Correlation between experimental ΔG and predicted binding energy obtained from screening (scr) MM-PBSA and standard (std) MM-PBSA of s_mmpbsa and standard (std) MM-PBSA of g_mmpbsa.

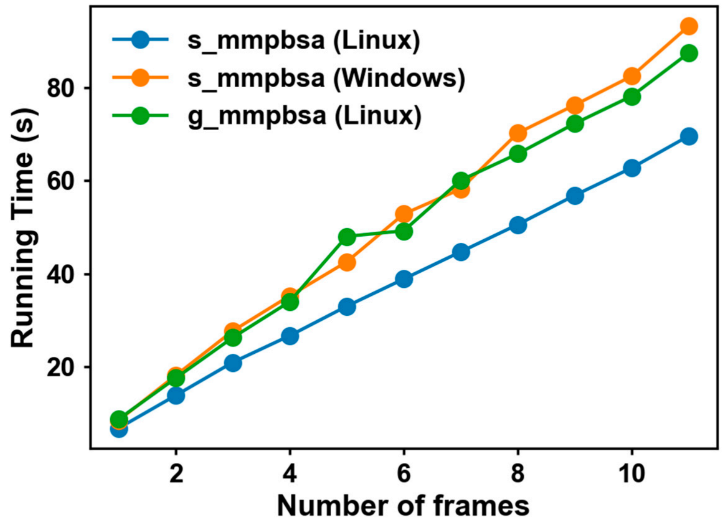

Figure S2 Cost of running time comparison of s_mmpbsa and g_mmpbsa on 1AJV system across different numbers of calculated frames.

Tables

Table S1 Different atomic radii sets s_mmpbsa used. "*" represents other atom types.

Atom Name [a]	Amber	Bondi	mBondi	mBondi2
HC	1.3	—	—	—
HN	—	—	—	1.3
HO	0.8	—	—	—
HS	0.8	—	—	—
HP	—	—	—	—
HA [b]	—	—	1.0	—
H	1.2	1.2	1.2	1.2
CA [c]	—	—	1.7	—
C	1.7	1.7	1.7	1.7
N	1.55	1.55	1.55	1.55
O	1.5	1.5	1.5	1.5
F	1.5	1.5	1.5	1.5
Si	2.1	2.1	2.1	2.1
P	1.85	1.85	1.85	1.85
S	1.8	1.8	1.8	1.8
Cl	1.7	1.7	1.7	1.7
Br	1.85	1.85	1.85	1.85
I	1.98	1.98	1.98	1.98
*	1.5	1.5	1.5	1.5

[a] Second alphabet after H represents the connected element.

[b] Aromatic hydrogen.

[c] Aromatic carbon.

Table S2 The PDB entries and inhibition constants (K_i) (in nM) of the 37 HIV-1 protease inhibitor complexes.

PDB ID	K_i	PDB ID	K_i	PDB ID	K_i
1EC2	0.1	1W5X	4	2PSV	58
1D4H	0.1	1D4J	4.4	2QNN	70
1EBZ	0.4	2CEN	5	2UY0	120
2AQU	0.48	1W5V	7.1	2PWR	260
1EBW	0.9	1G35	7.3	2PWC	270
1EC3	0.92	2BQV	9	2QNP	390
1EC1	1.2	1G2K	11	2QNQ	770
1T7K	1.37	2CEM	12	3BGB	900
1D4I	1.4	1AJX	12.2	1XL2	1500
2CEJ	2.4	1AJV	19.9	2PQZ	2150
1EC0	3.2	1IZH	20	3BGC	9600
2UXZ	3.3	2PSU	24		
1W5Y	3.3	1XL5	45		

Table S3 Comparison of binding energy for 37 HIV-1 complexes using s_mmpbsa and g_mmpbsa and experimental data.

	ΔG (exp)	ΔH (s_mmpbsa_std)	ΔH (s_mmpbsa_scr)	ΔH (g_mmpbsa_std)	ΔG (s_mmpbsa_std)	ΔG (s_mmpbsa_scr)
1AJV	–57.080	–167.185±4.095	–163.263±3.699	–167.554±4.097	–123.139±4.465	–125.758±4.790
1AJX	–57.080	–160.844±0.996	–157.710±0.980	–160.413±1.165	–130.262±6.737	–132.772±6.981
1D4H	–53.644	–162.470±7.891	–153.740±8.349	–161.123±7.507	–103.344±17.312	–111.563±17.492
1D4I	–53.192	–197.799±9.409	–189.225±9.070	–197.821±9.656	–134.929±17.168	–142.158±17.020
1D4J	–51.633	–173.779±8.360	–167.937±8.114	–172.983±8.309	–128.837±12.619	–133.938±12.674
1EBW	–51.579	–139.756±8.790	–131.733±8.262	–139.072±8.301	–84.558±13.995	–91.546±12.613
1EBZ	–50.920	–193.008±9.446	–187.339±9.574	–191.979±9.174	–142.042±26.262	–149.007±26.155
1EC0	–50.592	–225.550±0.429	–215.110±0.718	–225.111±0.909	–169.142±10.988	–179.719±10.307
1EC1	–50.538	–194.087±6.603	–188.261±7.608	–192.529±7.342	–134.092±7.003	–143.048±4.465
1EC2	–49.202	–175.905±2.273	–168.332±2.807	–173.832±1.282	–107.461±15.391	–113.892±15.409
1EC3	–48.489	–207.418±9.627	–203.528±9.967	–206.250±9.368	–168.762±14.083	–172.171±13.537
1G2K	–48.412	–167.635±1.843	–160.671±1.653	–167.330±2.285	–109.951±5.575	–115.571±5.941
1G35	–48.412	–172.403±12.455	–165.022±12.179	–172.233±12.408	–118.332±7.386	–124.459±7.369
1IZH	–47.936	–166.678±8.395	–163.363±9.007	–165.790±8.404	–122.271±12.731	–124.523±11.804
1T7K	–47.699	–146.776±5.976	–143.097±6.460	–146.103±5.870	–106.842±3.293	–109.690±4.232
1W5V	–47.382	–189.721±5.222	–183.753±5.298	–189.959±4.759	–144.279±6.661	–150.563±6.545
1W5X	–46.513	–200.387±5.485	–193.895±6.083	–200.542±5.561	–152.862±12.942	–158.776±11.797
1W5Y	–46.444	–225.011±1.676	–215.263±1.428	–224.013±1.464	–170.342±10.823	–179.929±10.564
1XL2	–45.925	–158.499±4.063	–157.299±4.367	–158.573±4.090	–127.967±8.462	–129.364±7.440

Table S3 Comparison of binding energy for 37 HIV-1 complexes using s_mmpbsa and g_mmpbsa and experimental data. (continued)

	ΔG (exp)	ΔH (s_mmpbsa_std)	ΔH (s_mmpbsa_scr)	ΔH (g_mmpbsa_std)	ΔG (s_mmpbsa_std)	ΔG (s_mmpbsa_scr)
1XL5	–45.428	–146.506±5.223	–139.829±4.610	–149.525±5.409	–111.170±7.703	–117.757±8.131
2AQU	–45.212	–197.845±1.258	–195.015±1.278	–196.893±1.295	–156.958±5.567	–158.589±4.962
2BQV	–45.171	–165.590±10.486	–162.276±10.598	–164.780±10.750	–122.743±20.494	–126.519±19.270
2CEJ	–43.958	–177.080±7.317	–174.130±6.733	–179.185±7.443	–119.489±17.511	–120.706±17.591
2CEM	–43.946	–182.754±6.185	–179.157±6.463	–181.632±5.680	–142.336±2.218	–145.584±2.217
2CEN	–43.494	–165.960±24.907	–163.217±24.519	–164.365±24.558	–121.650±24.890	–124.059±25.303
2PQZ	–41.936	–129.091±7.105	–127.178±7.553	–127.963±7.079	–74.695±18.788	–75.629±18.895
2PSU	–41.306	–137.172±10.551	–135.461±10.491	–137.217±10.263	–96.417±8.488	–97.233±7.670
2PSV	–40.840	–121.334±8.146	–117.342±9.251	–121.652±8.659	–56.655±20.065	–58.621±19.679
2PWC	–39.504	–92.790±8.091	–89.949±8.053	–91.917±8.252	–49.819±2.673	–51.533±2.685
2PWR	–37.587	–97.369±8.253	–97.374±8.032	–96.988±8.558	–63.032±8.141	–62.433±6.974
2QNN	–37.494	–112.380±6.152	–110.486±7.743	–110.941±6.094	–58.340±10.205	–58.223±10.001
2QNP	–36.582	–161.177±8.040	–153.085±7.258	–159.783±8.299	–123.761±4.169	–130.518±5.585
2QNQ	–34.896	–134.574±10.977	–129.971±9.226	–134.214±11.134	–81.864±6.540	–81.508±5.885
2UXZ	–34.509	–178.830±4.091	–175.703±3.582	–182.142±3.891	–132.947±11.128	–134.977±11.155
2UY0	–33.243	–185.427±3.697	–182.354±3.897	–187.746±4.450	–138.771±8.892	–141.337±9.406
3BGB	–32.350	–118.703±7.261	–111.434±6.206	–118.542±7.286	–80.203±9.393	–86.956±11.299
3BGC	–28.641	–120.878±9.985	–113.676±10.095	–120.470±9.990	–78.375±8.013	–84.100±8.047

Table S4 The parameters used in ΔG_{polar} calculation.

	s_mmpbsa	g_mmpbsa
cfac	1.5	1.5
gridspace	0.5	0.5
fadd	5	5
pconc	0.15	0.15
nconc	0.15	0.15
pcharge	1	1
ncharge	−1	−1
prad	0.95	0.95
nrad	1.81	1.81
pdie	2	2
sdie	78.4	78.4
vdie	1	1
srad	1.4	1.4
chgm	spl4	spl4
srfm	smol	smol
swin	0.30	0.30
sdens	10	10
temp	298.15	298.15
bcfl	mdh	mdh
PBsolver	lpbe	lpbe

Table S5 The parameters used in Δ$G_{\text{non-polar}}$ calculation.

	s_mmpbsa	g_mmpbsa
gamma	0.0226778	0.0226778
srad	1.4	1.4
sasaconst	3.84982	3.84982
sdens	10	10
dpos	0.2	0.2
grid	0.1 0.1 0.1	0.1 0.1 0.1
srfm	sacc	sacc
swin	0.3	0.3
temp	298.15	298.15
press	0	0

References

1. Ding, H.; Yin, Y.; Ni, S.; Sheng, Y.; Ma, Y., Accurate Evaluation on the Interactions of SARS-CoV-2 with Its Receptor ACE2 and Antibodies CR3022/CB6. *Chin. Phys. Lett.* **2021,** *38*, (1), 018701. DOI: 10.1088/0256-307X/38/1/018701
2. Case, D. A.; Cheatham III, T. E.; Darden, T.; Gohlke, H.; Luo, R.; Merz Jr., K. M.; Onufriev, A.; Simmerling, C.; Wang, B.; Woods, R. J., The Amber biomolecular simulation programs. *J. Comput. Chem.* **2005,** *26*, (16), 1668–1688. DOI: 10.1002/jcc.20290
3. Bondi, A., van der Waals Volumes and Radii. *J. Phys. Chem.* **1964,** *68*, (3), 441–451. DOI: 10.1021/j100785a001
